# A Fiber Bragg Grating Sensor Based on Cladding Mode Resonance for Label-Free Biosensing

**DOI:** 10.3390/bios13010097

**Published:** 2023-01-06

**Authors:** Shimeng Chen, Chao Zhang, Jiahui Wang, Na Li, Yongxin Song, Haojun Wu, Yun Liu

**Affiliations:** 1 Department of Marine Engineering, Dalian Maritime University, Dalian 116026, China; 2 School of Physics, Dalian University of Technology, Dalian 116024, China

**Keywords:** fiber Bragg grating, biosensor, label-free, limit of detection, graphene oxide

## Abstract

A fiber-optic biosensing platform based on ultra-narrowband cladding mode resonances was developed on a high-reflectivity fiber Bragg grating (FBG) for targeting biomolecular detection. The multiple cladding modes with a high sensitivity to the refractive index (RI) were excited in the FBG by coupling between the forward-propagating guided core mode of the multimode fiber and the backward-propagating guided cladding mode of the FBG without any damage to the fiber structure or any change to the standard FBG manufacturing process. The full width at half maximum and the Q-factor of the typical cladding mode resonance operation of the proposed sensor are 80 pm and 19,270, respectively, which are better than those of most fiber-optic biosensors reported to date. In addition, the FBG sensor demonstrated a high sensitivity in protein detection and a high selectivity in serum sample assays. The sensitivity of this sensor was further increased simply by coating it with graphene oxide (GO) sheets on the sensing surface without using a signal amplification strategy. Furthermore, an ultra-low limit of detection (LOD) of 32 pM was obtained by the GO-coated FBG sensor for IgG detection. The proposed FBG sensor provides a competitive fiber-optic platform for biomolecular detection. It has a great potential for applications in label-free biosensing.

## 1. Introduction

At present, the development of label-free fiber-optic biosensors is expanding at a faster than expected pace [1,2]. A label-free fiber-optic detection technique can be used for kinetic analysis and quantitative detection of specific analyte to probe binding events while maintaining the biological functions of proteins [3,4]. Moreover, it is advantageous in many aspects, such as low cost, excellent biocompatibility, easy miniaturization, good flexibility, high sensitivity, etc. [5,6,7,8]. For most label-free fiber-optic biosensors, the optical fiber device is used as a transducer functionalized with a biological layer on the surface. The biological layer contains a biological recognition element, whose selectivity to the well-defined target helps to improve the capability of biological binding to the target. By converting the interaction of the biological layer with the target biomolecules into optical signals, highly sensitive and selective detection is achieved using the optical fiber devices. Currently, various optical fiber sensors are increasingly being used for label-free biosensing. Among them, the most common fiber biosensors are all-fiber interference devices, including the Mach–Zehnder interferometer (MZI), Michelson interferometer, Fabry–Perot interferometer (FPI), and multimode interferometer [9,10,11,12]. To achieve the visibility of a fiber interferometer, one popular method is to fuse different fibers into a hetero-core structure [13,14,15] where light from the input fiber is coupled onto lots of high-order modes in the inserted fiber due to the core mismatching. Some of these modes are re-coupled to interfere with each other in the output fiber. As a result, several transmission and attenuation bands are observed in the spectrum corresponding to the destructive structural interference. Another approach to generating an interference spectrum is to change the shape of the transmission fiber by using femtosecond laser ablation, non-adiabatic tapering, or side polishing [16,17,18,19]. However, reproducing the fiber shape to the desired structure always leads to the fragility of a fiber sensor. Furthermore, it is also challenging to maintain consistency in the manufacture of these sensors.

Fiber gratings are other types of commonly used fiber biosensing devices, such as etched or side-polished fiber Bragg gratings (FBGs), long-period fiber Bragg gratings (LPG), and tilted fiber Bragg gratings (TFBG) [20,21,22]. For these fiber grating devices, the effective RI of the cladding mode is affected by the biomolecules immobilized on the fiber surface. This can be detected by monitoring the changes in the resonance wavelength. FBGs, the most mature passive optical fiber devices amongst the fiber grating devices, are widely used in the fields of fiber-optic communications and fiber-optic sensing [23,24,25,26]. However, one shortcoming of FBGs is that they are insensitive to the change in the surrounding refractive index (RI). In order to use FBGs for biosensing, it is necessary to dramatically reduce their cladding diameter to improve RI sensitivity, either by corroding or by polishing [27,28]. However, the cladding removal process inevitably makes FBGs highly fragile. Unlike FBGs, TFBGs have the RI perturbation planes that are tilted with respect to the fiber axis. This makes it possible to excite the back-propagating cladding modes to reach the interface between the cladding and the external medium [29,30,31]. Accurate measurements of the external RI can be achieved by monitoring the wavelength or amplitude variations of the cladding mode resonances. Like TFBGs, the resonance wavelength in LPFGs also depends on the cladding mode and the RI. The RI perturbations in the core of LPFGs are long periodic. This enables the coupling between the core mode and the cladding mode to propagate in the same direction [32,33]. To improve the RI sensitivity of LPFGs, one approach is to coat the cladding surface with a thin layer of high RI (HRI) material. This enables the LPFG sensor to work in the mode transition region [34,35]. While both TFBG and LPG sensors are sensitive to changes in RI and have good stability, their fabrication costs are typically much higher than those of FBGs due to the differences in fabrication processes and the limitations in commercial availability.

Motivated by the realization of low-cost and high-performance fiber-optic biochemical monitoring, we proposed a high-reflectivity FBG biosensor based on ultra-narrowband cladding mode resonances in this paper. The transmission spectrum of the sensing structure shows a dense comb-like resonance due to the coupling between the forward-propagating guided core mode of the multimode fiber (MMF) and the backward-propagating guided cladding mode of the FBG. The narrow-band resonance possesses the narrow full width at half maximum (FWHM). This leads to a high Q-factor. The resonant wavelengths of cladding modes are sensitive to the RI and exhibits high stability in the blank buffer sample. As a typical demonstration, the FBG sensor was functionalized with anti-IgG for the detection of IgG at different concentrations and achieved the excellent limit of detection (LOD). The specificity of the FBG sensor was also verified by detecting IgG in serum. After that, we chose the graphene oxide (GO) sheet as the sensitizing material of our proposed FBG sensor to further improve the sensitivity. The GO film as a high RI film leads to enhanced evanescent waves. Its functional group promotes the bio-functionalization of the sensor through covalent bonding. Finally, the GO-coated FBG sensor achieves a high sensitivity and an ultra-low LOD, which is an order of magnitude lower than that of an uncoated FBG sensor. The aim of this work is to demonstrate an FBG as a label-free sensing platform as a proof of concept. It features simplicity, low cost, and high performance compared to the currently reported optical fiber configuration.

## 2. Materials and Methods

### 2.1. Materials and Reagents

GO dispersion was purchased from Nanjing XFNANO Materials Tech Co., Ltd. (Jiangsu, China); (3-Aminopropyl) triethoxysilane (APTES) was purchased from Tokyo Chemical Industry Co., Ltd. (Shanghai, China); 1-(3-Dimethylamino-propyl)-3-ethylcarbodiimide hydrochloride (EDC) and N-Hydroxysuccinimide (NHS) were purchased from Saan Chemical Technology Co., Ltd. (Shanghai, China); immunoglobulin G (IgG) from rabbit serum, anti-rabbit IgG antibody produced in goat, rabbit serum, and phosphate buffer solution (PBS, pH 7.4) were purchased from Sangon Biotech Co., Ltd. (Shanghai, China); and bovine serum albumin (BSA), glutaraldehyde, and other chemicals were purchased from Sigma-Aldrich Trading Co., Ltd. (Shanghai, China). All of the reagents were of analytical grade and could be used without further purification. All proteins were dissolved into PBS before use.

### 2.2. Antibody Immobilization

The sensing probe was first cleaned using piranha solution (1:3 vol ratio H_2_SO_4_: H_2_O) at 80 °C for 30 min. Subsequently, the sensing probe was mixed with 5% APTES in acetone and further incubated for 30 min. The sensing probe was further reacted in 25% glutaraldehyde at 80 °C for 30 min. After glutaraldehyde fixation and cleaning, the sensing probe was placed in anti-IgG solution (0.1 mg/mL) for 60 min. Thereafter, the sensing probe was soaked in BSA solution (1 mg/mL) as a blocking reagent for 15 min. In each case, the sensing probe was cleaned and dried with deionized water and N_2_ in turn.

### 2.3. GO Deposition and Antibody Immobilization

The first two steps were the same as the ones in the last section. After APTES solution treatment, the sensing probe was reacted in GO dispersion (1 mg/mL) overnight, and the GO sheet was self-assembled on the sensing surface. Subsequently, the sensing probe was reacted in NHS/EDC (0.5/0.5 M) mixed solution at 4 °C for 20 min. Anti-IgG solution (0.1 mg/mL) was injected onto the sensing probe for 60 min to attach the sensing probe. Finally, the sensing probe was soaked in BSA solution (1 mg/mL) for 15 min. In each case, the sensing probe was cleaned and dried with deionized water and N_2_ in turn.

### 2.4. Sensor Preparation and Interrogation

**Figure 1:**
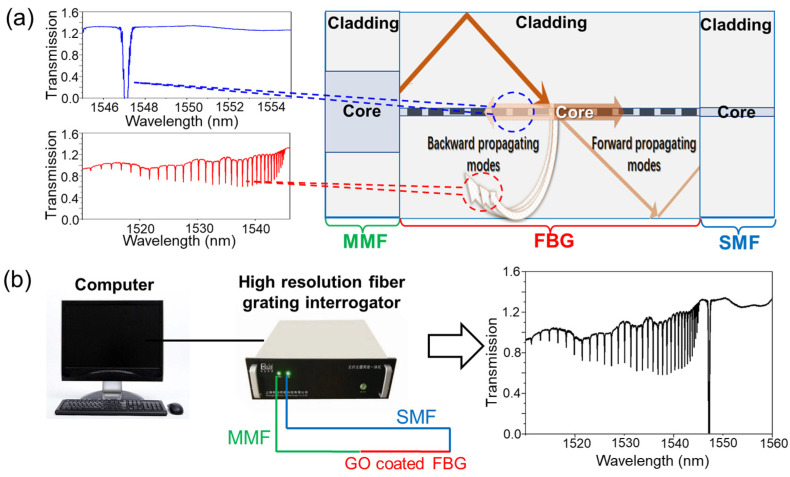
Illustrations of the sensing element (**a**) and the sensing system (**b**) of the FBG sensor based on cladding mode resonance.

Figure 1 shows the sensing element and the sensing system of the FBG sensor that we design in this paper. As shown in Figure 1a, the sensing element is composed of a high reflection FBG, graded-index MMF, and single-mode fiber (SMF). An FBG with a length of 4 mm was fabricated through the phase mask method using SMF-28e (no hydrogen carrying process). It achieved an ultra-high reflectivity, greater than 99.9% through a photo-inscription process over a long time (15 min). One side of this FBG that was close to the grating area was cut to splice with an MMF with core/cladding of 62/125 μm diameters. Figure 1a illustrates the cladding modes coupling when the light from the MMF was diffracted by grating in the FBG, whose insets are the transmission spectra of backward propagating modes and forward propagating modes. In our work, all transmission spectra of the sensor were measured by a high resolution fiber grating interrogator, as shown in Figure 1b. Being the input and output fibers, the MMF and SMF are connected to the fiber grating interrogator using FC/PC connectors. The transmission spectra of our sensor were reported by a computer based on the LabVIEW platform, as shown in the inset of Figure 1b. The interrogation system has a high resolution of 4 pm and a high scan frequency of 5 kHz.

## 3. Results and Discussion

### 3.1. Sensing Characteristics of the Sensing Probe

**Figure 2:**
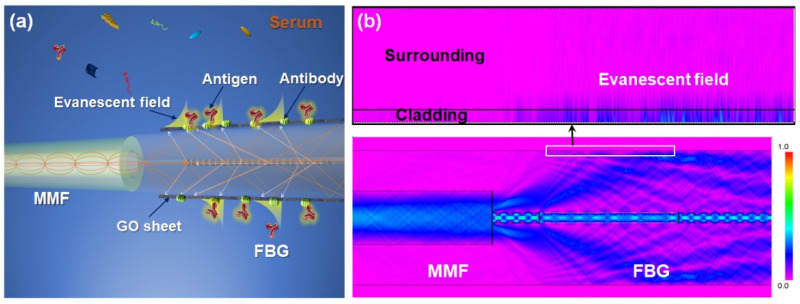
(**a**) The coupling between the optical fiber modes mediated by the RI perturbation caused by protein molecule specific binding. (**b**) The simulated optical field distribution in the FBG.

The schematic overview of the FBG sensor is shown in Figure 2a. The FBG sensor consists of a graded-index MMF and a high-reflectivity FBG based on a standard single-mode fiber. In the FBG structure, there are forward and backward propagation modes due to the existence of periodic RI modulation regions in the core [36]. Furthermore, since the incident light originates from an MMF, it can excite strong cladding modes after passing through the FBG grating region. The evanescent filed on the fiber surface was simulated by Rsoft software (Lighttec). The simulated optical field distribution in the FBG is shown in Figure 2b. The grating period is set as 0.532 μm. The peak reflectivity of the grating is set as 100% and the grating length is set as 4 mm. Simulation results show that the backward cladding modes are excited in the grating region and interact with the surroundings via the evanescent field. The coupled-mode theory can also prove our simulation result [37].

**Figure 3:**
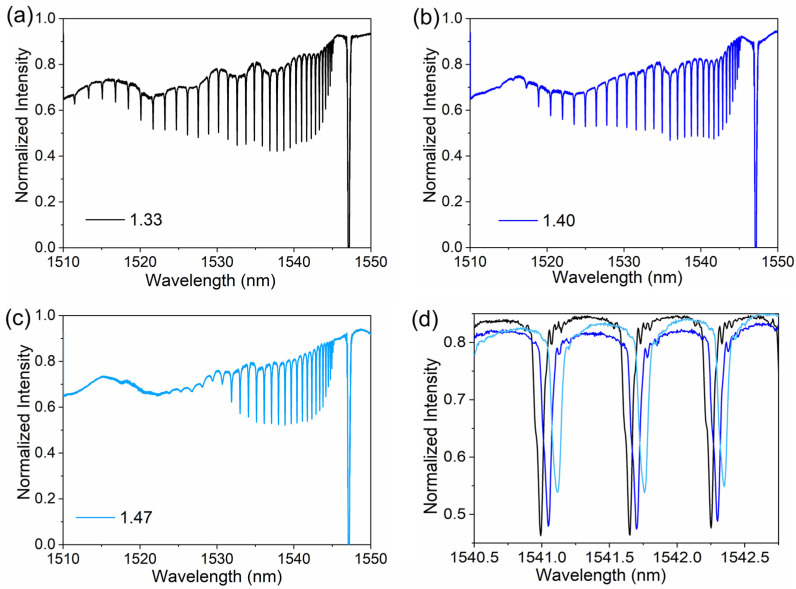
Experimental transmission spectra through a high-reflectivity FBG in the RI of 1.33 (**a**), 1.40 (**b**), and 1.47 (**c**). (**d**) Enlarged drawing of the narrow FWHM of the FBG sensor.

The experimental transmission spectra of the FBG sensor in different RI environments, obtained by using the sensing system described in Section 2.4, are shown in Figure 3. The experimental spectrum shows a dense comb-like narrow-band resonance. This also indicates the mode-coupling behavior in the FBG region. The external medium is deionized water with a lower RI than that of the fiber, and the transmission loss of the cladding mode on the grating is small. Thus, the coupling among the different cladding modes gives rise to a clear resonance in the transmission spectrum. Due to the high reflectivity of the FBG and the forward cladding modes excited by the MMF-FBG structure, the number of strong backward propagating cladding modes can be excited by the fundamental mode and forward cladding modes interacting with the grating region. Since the cladding modes have various effective RIs slightly smaller than those of the core modes, the dips corresponding to the backward propagating cladding modes in the transmission spectrum are in turn located on the side of the short wavelength range relative to the Bragg mode of the fiber core. In addition, in the experimental transmission spectrum, the Bragg mode (core mode) possesses the ultra-high reflectivity, meanwhile, backward propagating cladding modes have the ultra-narrow FWHM. As the RI increases, the sharp cladding mode resonances in the short wavelength range become shallower, or even disappear, due to the appearance of the leaky cladding modes (radiation mode). It has been shown that the cladding mode resonance is highly sensitive to RI, indicating the potential of this FBG sensor for biosensing applications.

For the optical sensors that use the resonance dip as the sensing signal, the Q-factor associated with the FWHM is very important for the sensing resolution. The FWHMs of resonance dips at short wavelengths are significantly affected by the RI of the surrounding medium. However, the RI of the surrounding medium has little effect on the FWHMs of resonance dips at long wavelengths. The difference in FWHM is less than 5 pm between 1.33 and 1.47 for the surrounding medium. The FWHM of the resonance dips within 1535–1545 nm does not exceed 80 pm Although some resonance dips have FWHM as narrow as 50 pm, we still use 80 pm as a performance indicator for our sensor. We chose the resonance dip at 1541.6 nm as the test dip, as shown in Figure 3d. The Q-factor is calculated as 19,270 from the ratio of the central wavelength to the FWHM. It is higher than that of most types of fiber-optic biosensors, such as the LSPR fiber sensor (Q-factor~6, [38]); fiber FPI (Q-factor~62, [10]); LPFG sensor (Q-factor~106, [39]); SPR fiber sensor (Q-factor~135, [40]); etched fiber sensor (Q-factor~138, [41]); fiber MZI (Q-factor~153, [9]); and TFBG sensor (Q-factor~7022, [42]). The Q-factor verifies that the proposed biosensor has a great potential in detecting many biomolecules for which conventional optical fibers are not sensitive enough.

### 3.2. Protein Detection Using the Sensing System

**Figure 4:**
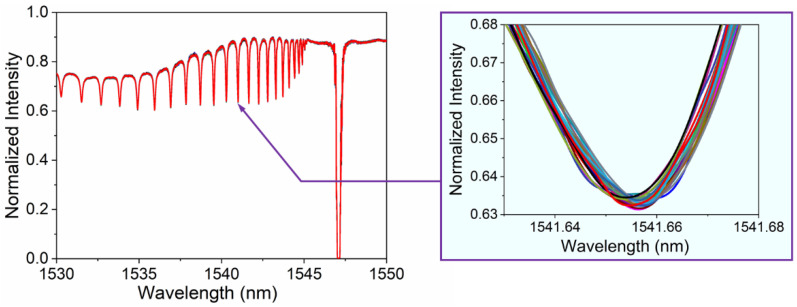
Spectral change of cladding mode resonance near 1541.6 nm for 50 min in buffer.

Before conducting biochemical sensing, we investigated the stability of the FBG sensor and the error in the dip detection. We placed the sensor probe in the PBS buffer and monitored the spectra for 50 min (the spectra were recorded every 2 min). The experimental data were collected using a computer program developed by LabVIEW. A total of 25 sets of spectral data results were obtained and plotted, as shown in Figure 4. It can be found that the resonant wavelength shows little variation during the 50 min of PBS soaking, indicating excellent stability in biochemical sensing.

**Figure 5:**
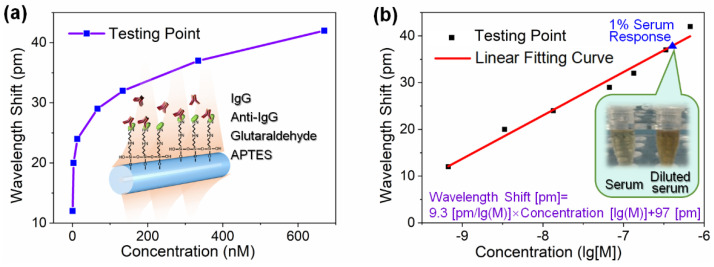
(**a**) The wavelength response of the cladding mode resonance increases with IgG concentration. Inset: schematic illustration for the functionalization of the FBG sensor with an anti-IgG aptamer and an IgG detection kit. (**b**) Wavelength response as a function of the IgG concentration on a logarithmic scale.

In order to test the biosensing capabilities of our sensors, the FBG sensors were applied for the detection of bioreceptor-analyte complexes such as anti-IgG/IgG. We injected rabbit IgG samples at different concentrations, and the samples would then flow over the sensing surface modified with goat anti-rabbit IgG. The resonant wavelength shift around 1541.6 nm was used as the sensor response. The wavelength shifts were recorded in real-time when the IgG was bound to anti-IgG during the test. The specific binding responses were stabilized within 10 min, indicating the successful binding of IgG to anti-IgG at the sensing surface. After one round of IgG testing, we injected urea into the flow cell to regenerate the sensing surface. Thereafter, we proceeded to the next sample test. Following the above procedure, we successively assayed the IgG samples at the concentrations of 670 nM, 335 nM, 134 nM, 66.7 nM, 13.4 nM, 3.35 nM, and 0.667 nM. The result is shown in Figure 5a. More antibodies were immobilized on the surface of the biosensor as the IgG concentration increased, leading to a shift of the resonant wavelength towards longer wavelengths. We recorded the wavelength response of the 1541.6 nm resonance at each concentration. We plotted the relation between the wavelength shift versus concentration on a logarithmic scale. Thus, we can curve fit the experimental datum points in Figure 5b and obtain the functional relation as: wavelength shift [pm] = 9.3 concentration (lg[M]) + 97[pm]. Based on this relation, the theoretical LOD was calculated using the method recommended by IUPAC in the Journal of Analytical Chemistry. Considering the non-specific adsorption at 6 pm and the triple standard deviations of 3.6 pm, the theoretical LOD can be calculated as 360 pM.

In addition, in order to evaluate the specificity of our sensor for samples with complex compositions, we also used the sensor to detect IgG in diluted rabbit serum. In this experiment, we chose a serum concentration of 1 percent, which corresponds to the linear calibration range of the sensor. The response of the sensor to the rabbit serum was approximately 38 pm, corresponding to a concentration of 417 nM. This means that the concentration of IgG in the serum is predicted to be about 6.3 mg/mL which ranges from 5 to 12 mg/mL for normal rabbit serum. This result confirms that our sensor has good accuracy and high selectivity to IgG when thousands of other proteins are present in the serum.

### 3.3. GO-HRI Layer and Sensitivity Enhancement

**Figure 6:**
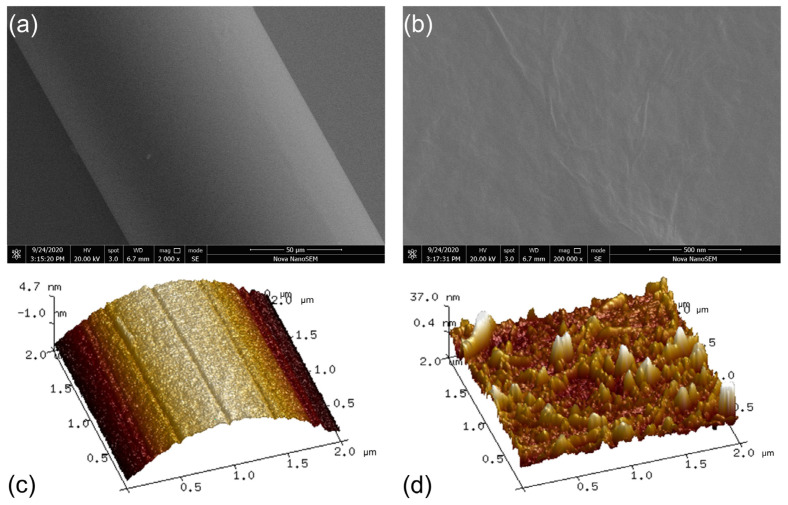
(**a**) Scanning electron microscope analysis of the GO-coated FBG sensor. (**b**) Observation of the GO-coating on the cladding surface. Atomic force microscope analysis of FBG sensor before (**c**) and after (**d**) GO-coating.

**Figure 7:**
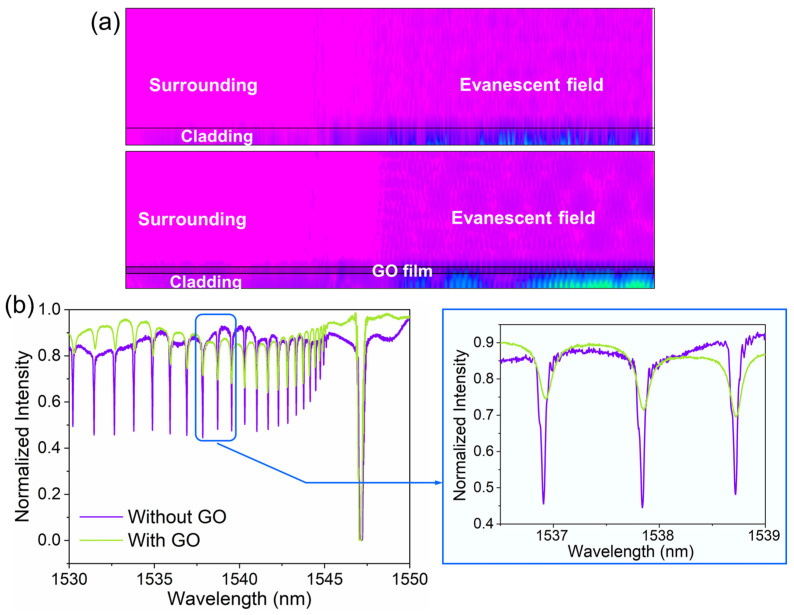
(**a**) The simulated optical field on the cladding surface of the FBG before and after GO-coating. (**b**) The comparison in the transmission spectrum of the FBG sensor before and after GO-coating.

To achieve a higher sensitivity in label-free biosensing, the FBG sensor was designed with an overlay to tune the evanescent field on the cladding surface. In our work, GO sheets were selected to coat the cladding as an HRI material. This could enable the formation of a uniform thin layer on the fiber surface via dip-coating deposition method. The use of GO film-based HRI coatings leads to a reorganization of the cladding mode. It allows higher-order cladding modes to propagate over the overlayer. This leads to a larger shift of the corresponding resonance wavelengths and a higher contribution of their evanescent waves. In addition, GO exhibits good biocompatibility and high affinity for specific biomolecules due to hydrophilic oxygen containing functional groups [43]. The carboxyl functional group of GO was converted to activated ester by NHS/EDC mixed solution, which can react with the amino group of the protein molecule. Due to the functional groups of the GO sheets, the GO layer may promote the biofunctionalization of FBG sensors. The GO-coated FBG was analyzed by scanning electron microscope (Nova NanoSEM 450), as shown in Figure 6a,b, and the atomic force microscope (Dimension Icon, Bruker, Germany), as shown in Figure 7a,b. It can be seen that GO adheres to the surface of the cladding of the FBG and forms a compact rough surface after completion of the coating process.

We investigated the interaction of the GO layer with the evanescent field by simulation, as shown in Figure 7a. Because of the appearance of GO, the intensity of the optical field increases significantly above the GO layer. This can be explained as follows: the amplitude enhancement factor of the evanescent wave excited on the surface of the cladding can be expressed as ${\vec{E}}_{0}\exp\left[-\frac{2\pi }{\lambda }z\sqrt{{\left(\frac{\sin i}{n_{21}}\right)}^{2}-1}\right]$ [44]. Here, ${\vec{E}}_{0}$ represents the incident light wave. λ and i are the wavelength and the incident angle.$\;n_{21}$ is the relative RI of the fiber to the external environment. z is the vertical distance away from the fiber surface. When the GO film is used as the HRI layer covering the cladding, the critical incidence angle of the total reflection decreases and the relative RI $n_{21}$ increases. This results in an increase of the amplitude enhancement factor of the evanescent wave. Figure 7b shows a comparison in transmission spectrum of the FBG sensor before and after coating with GO sheets. Since the difference in RI between the core and the cladding layer is reduced, the coupling coefficient associated with the cladding modes in one fiber loop decreases with increasing RI. Thus, we can find that the cladding mode resonance dip becomes significantly shallower after GO-coating. The loss ratio decreases as the wavelength increase of the cladding mode resonance dips, while the FWHM becomes wider. Overall, we still chose the cladding mode resonance dip at 1541.6 nm as the testing dip in order to achieve a great sensing performance. Its loss ratio is approximately 45%.

**Figure 8:**
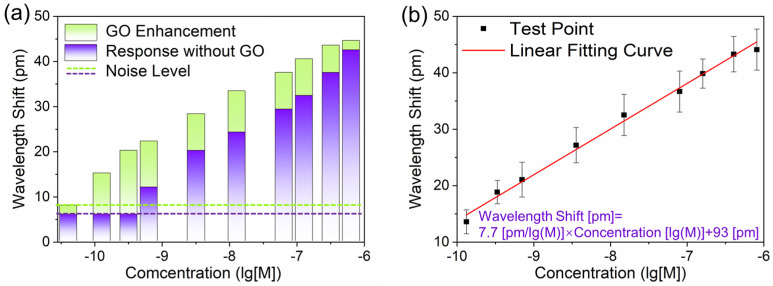
(**a**) The comparison in wavelength response of the FBG sensor to different IgG samples before and after GO-coating. (**b**) Wavelength shift of the GO-coated FBG sensor as a function of the IgG concentration on a logarithmic scale.

We performed a functionalization of the GO-coated FBG sensor and applied it to the detection of IgG at different concentrations. The responses with and without GO-coating at each concentration of the FBG sensor are compared in Figure 8a. As can be seen from the comparison, the GO sheets on the cladding surface can significantly enhance the sensor response, especially for the IgG sample with a low concentration. Moreover, the GO-coated FBG sensor has an experimental detection limit of 134 pM, which is around a third of that of the FBG sensor without GO sheets. The relation between response and concentration on a logarithm scale is shown in Figure 8b. By using curve fitting, we calculated the functional relation as: wavelength shift [pm] = 7.7 concentration(lg[M]) + 93[pm]. Considering a non-specific adsorption of about 8 pm and the triple standard deviation of about 3.6 pm, we calculated and obtained a rather low LOD of 32 pM, which is an order of magnitude lower than that of the FBG sensor without GO coating.

In order to verify the reproducibility of our experimental results, the sensing chip was regenerated in high-concentration urea (8M) for 5 min. Each measurement at a given concentration was repeated three times. Variabilities of wavelength responses among the same concentration of IgG injections (n = 3) are estimated by relative errors (E_r_ are shown as error bars in Figure 8b). The standard deviations and relative standard deviations are not exceeding 3.51 and 14.26%, respectively. Hence, our sensor possesses good repeatability over a large detection range. To further verify the biosensing property of our sensor, a comparison between our work (the FBG sensor with and without GO-coating) and recently reported works based on label-free optical fiber sensors for IgG detection was performed (Table 1). It can be found that our sensor has a good ability to detect biomolecules and has a great potential for application in label-free optical fiber biosensing.

## 4. Conclusions

We demonstrate a high-reflectivity FBG biosensor based on ultra-narrowband cladding mode resonances. The proposed sensor is fabricated using the common fiber fusion splicing and fiber grating photo-inscription technologies. The FWHM of the typical cladding mode of the proposed sensor is 80 pm. Its Q-factor is as high as 19,270. Therefore, it is better than most optical fiber biosensors reported so far, including fiber interferometers, fiber grating sensors, and fiber SPR sensors. The sensor demonstrates a sensitive response to protein detection and a high selectivity in serum samples with complex protein composition. The thin layer of GO was introduced to achieve an improvement in sensitivity. Moreover, its high RI character effectively enhanced the excitation of the evanescent field. Without using signal amplification by functionalized gold nanoparticles or second antibodies, an ultra-low LOD of 32 pM was obtained by the GO-coated FBG sensor from IgG detection. This proves that our sensor shows a great potential for applications in highly sensitive and label-free biochemical sensing. In addition, the fabrication of the proposed high-reflectivity FBG biosensor involves only the standard FBG fabrication process and the optical fiber fusion technology. This ensures cost-effective mass production.

## Figures and Tables

**Table 1:** Comparison of the label-free optical fiber sensors for IgG detection.

Sensor	Molecule	LOD	Ref.
SPR image sensor	IgG	47.4 nM	[45]
TFBG-SPR sensor based on GO	IgG	0.5 μg/mL (3.35 nM)	[46]
Microcapillary-based LSPR sensor	IgG	3 nM	[47]
Bragg grating sensor based on tapered microfiber	IgG	0.1 μg/mL (667 pM)	[48]
FBG sensor based on cladding mode resonance	IgG	360 pM	**This work**
MZI sensor with large core-offset fusion splice	IgG	47 ng/mL (313 pM)	[49]
MZI sensor with S-tapered optical fiber	IgG	28 ng/mL (186 pM)	[50]
LPG sensor based on titania–silica thin film overlay	IgG	53 pM	[51]
FBG sensor based on cladding mode resonance with GO-coating	IgG	32 pM	**This work**

## Data Availability

Not applicable.
